# Iron Deficiency Anemia as a Rare Presentation of Subacute Bacterial Endocarditis Caused by a Rare Organism

**DOI:** 10.7759/cureus.74553

**Published:** 2024-11-27

**Authors:** Mohamed Abdulmajeed, Kunaratnam Thayaparan, Abdirashid Ali

**Affiliations:** 1 General Internal Medicine, Luton and Dunstable University Hospital, Luton, GBR

**Keywords:** bacterial endocarditis, cardiac complications, infective endocarditis, iron deficiency anemia (ida), streptococcus anginosus group, subacute bacterial endocarditis

## Abstract

Infective endocarditis commonly presents with fever, malaise, fatigue, and chest pain. However, this case report details an atypical presentation of infective endocarditis when a 63-year-old male patient was referred by his general practitioner to the emergency department with shortness of breath and substantial weight loss to investigate his symptomatic anemia. His initial assessments revealed severe iron deficiency anemia without any gastrointestinal or any other source of bleeding. Further investigations demonstrated that the patient suffered from infective endocarditis caused by a rare causative organism, *Streptococcus anginosus*. Hence, our case report highlights the atypical presentation and the rare organism in addition to the possibility of close association between iron deficiency anemia and subacute bacterial endocarditis. The patient was managed by culture-guided intravenous antibiotics and discharged after serial negative cultures.

## Introduction

Subacute bacterial endocarditis (SBE) usually results from an infection with *Streptococcus* species that are characterized by a low virulence and mild to moderate spectrum of clinical symptoms. The condition usually develops slowly over the course of more than two weeks at least [1]. Studies show that most of the patients with SBE usually present with fever, malaise, fatigue, and mild leukocytosis [2]. *Streptococcus anginosus* is part of a group called *Streptococcus milleri* that consists of *S. anginosus*, *S. intermedius*, and *S. constellatus*. *S. anginosus* was first isolated from a dental abscess by Guthof in 1956 [3]. They are usually non-pathogenic organisms that live normally in the human epithelium. However, if the host's immune system is compromised or the skin barrier or mucus membrane is disrupted, the organism finds access to the internal environment and turns into a pathogenic organism which causes multiple abscesses in different sites like the brain and maxillofacial, perianal, and soft tissue infections [3,4]. In our study, we presented a case report of a man with SBE secondary to *S. anginosus* whose main presentation was iron deficiency anemia (IDA) and progressive weight loss.

## Case presentation

A 63-year-old male patient was referred by his general practitioner to the emergency department after a diagnosis of IDA with a progressive course of shortness of breath, fatigue, loss of appetite, and weight loss (around 6 kg in one month) over the past two months. The patient denied any fever, a recent change in bowel habits (constipation or diarrhea), gastrointestinal bleeding (melena or hematemesis), lower limb edema, or palpitation. He had no past medical history of chronic conditions like diabetes mellitus, hypertension, or cardiac, respiratory, or gastrointestinal issues. Social history was significant for regular alcohol intake but no history of smoking or illicit drug use.

On examination, the patient appeared severely anemic with severe pallor most prominently on the face, lips, and conjunctiva. His vital signs were stable, with a blood pressure (BP) of 117/72 mmHg, a heart rate of 85 beats per minute (bpm), and an oxygen saturation at 95%. Cardiac auscultation revealed a systolic murmur best heard over the apex. Chest examination showed normal air entry with normal chest expansion and no adventitious sounds. Abdominal examination was normal with no hepatosplenomegaly.

**Figure 1:**
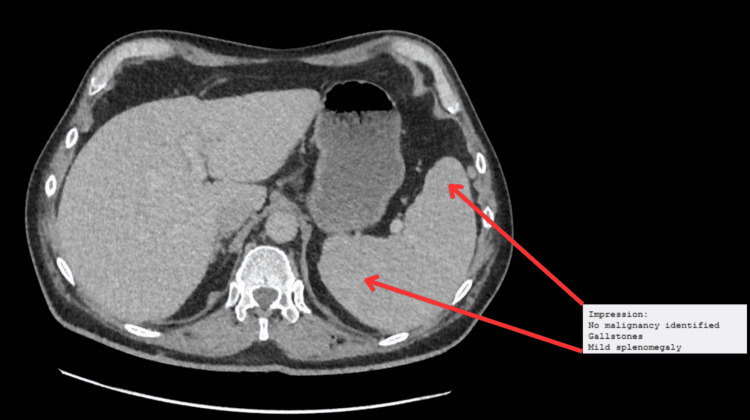
Mild splenomegaly in CT-CAP CT-CAP: computed tomography of the chest, abdomen, and pelvis

Laboratory results confirmed severe microcytic hypochromic anemia (hemoglobin: 74 g/L; mean corpuscular volume (MCV): 73) with reduced iron and transferrin saturation (Table 1). With the confirmed anemia, the patient reported weight loss along with loss of appetite which raised the suspicion of malignancy. Hence, a computed tomography of the chest, abdomen, and pelvis (CT-CAP) was ordered to rule out malignancy. The CT-TAP showed no signs of malignancy but showed the presence of mild splenomegaly (Figure 1).

**Table 1:** Laboratory test results WBC: white blood cell; RBC: red blood cell; MCV: mean corpuscular volume; MCH: mean corpuscular hemoglobin; MCHC: mean corpuscular hemoglobin concentration; RDW: red cell distribution width; NRBC: nucleated red blood cell; MPV: mean platelet volume

Parameter	Result	Unit	Reference range
WBC	5.4	10^9^/L	4.0-11.0
Hemoglobin	74	g/L	130-165
Platelets	183	10^9^/L	150-450
RBC	3.1	10^12^/L	4.5-5.9
Hematocrit	0.23	L/L	0.41-0.51
MCV	73	fL	80-100
MCH	24	pg	27-32
MCHC	326	g/L	280-355
RDW	15.8	%	11.8-14.8
Neutrophils	4.24	10^9^/L	2.0-7.0
Lymphocytes	0.70	10^9^/L	1.0-3.0
Monocytes	0.42	10^9^/L	0.2-1.0
Eosinophils	0.00	10^9^/L	0.0-0.4
Basophils	0.04	10^9^/L	0.02-0.1
NRBC	<0.5	10^9^/L	<0.5
MPV	7.5	fL	7.8-11.0

After careful consideration of the patient's complaints, symptoms, signs, and radiological findings, infective endocarditis was suspected, and a urine dipstick was requested as a bedside test to confirm microscopic hematuria, which can be a feature of infective endocarditis. The results of the urine dipstick confirmed the presence of microscopic hematuria.

The presence of cardiac murmur, microscopic hematuria, anemia, and mild splenomegaly should raise the suspicion of infective endocarditis as a differential diagnosis.

Cardiac murmur

This is due to the destruction of one or more heart valves.

Microscopic hematuria

This is common in infective endocarditis due to immune complex-mediated glomerulonephritis, which can cause kidney damage and lead to the presence of blood in the urine.

Anemia

Chronic infection, including endocarditis, often results in anemia of chronic disease. Additionally, anemia may result from hemolysis or bone marrow suppression due to inflammation.

Mild splenomegaly

This can occur due to the immune response to the persistent infection and the filtering of immune complexes and bacteria by the spleen.

**Figure 2:**
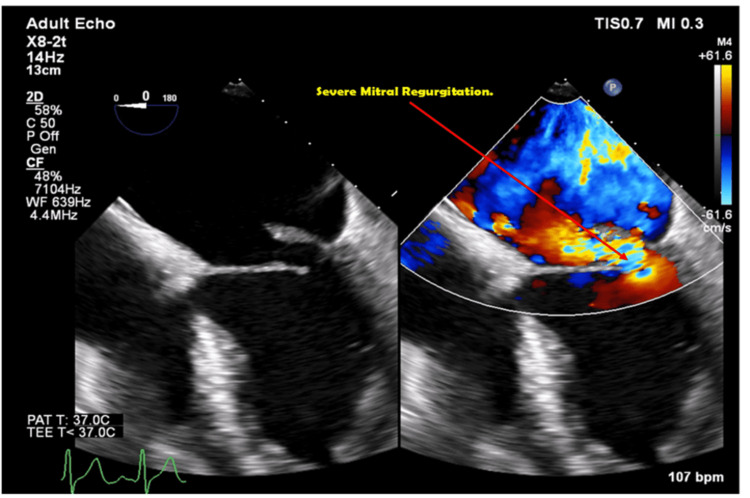
Severe mitral regurgitation on TEE TEE: transthoracic echocardiogram

**Figure 3:**
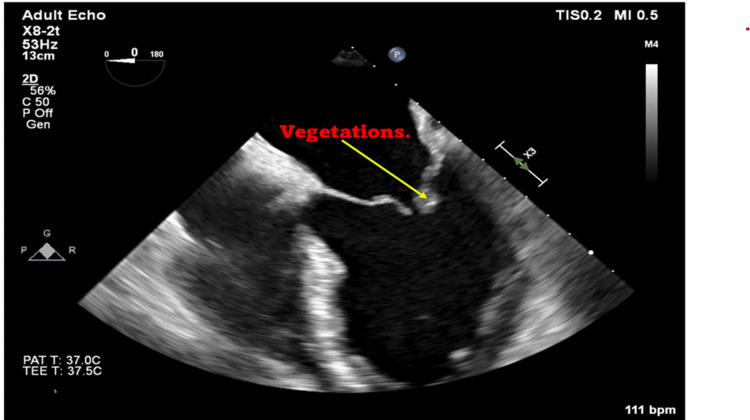
Mitral valve vegetations

**Figure 4:**
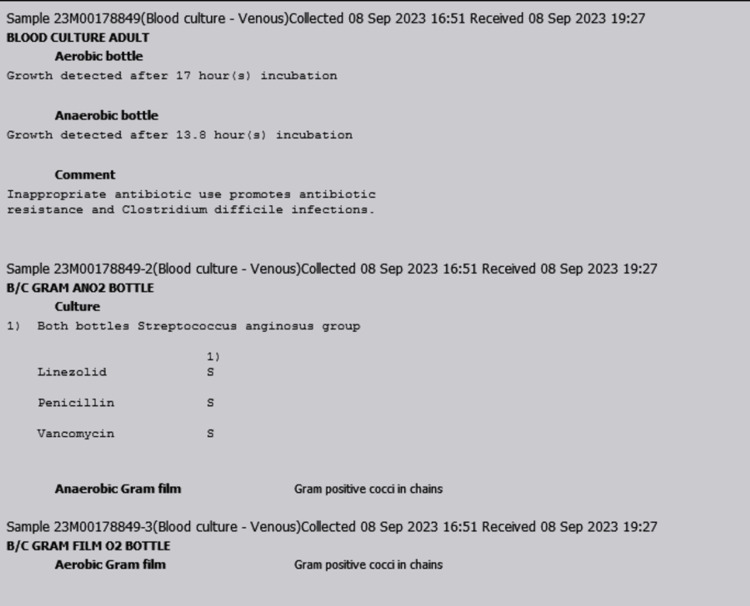
Blood culture

In this case, infective endocarditis was at the top of the differential diagnoses. Blood cultures, transthoracic echocardiogram (TTE), and transesophageal echocardiogram (TEE) were immediately ordered for the patient to exclude infective endocarditis. The TTE report revealed a mildly dilated left ventricle, dilated left atrium, flail mitral leaflets, severe mitral regurgitation, and mitral valve prolapse. The TEE confirmed the presence of posterior mitral valve leaflet prolapse and 15 mm vegetations attached to it (Figure 2 and Figure 3). Aerobic and anaerobic blood cultures were obtained from the patient and incubated from the microbiological activities, and aerobic and anaerobic bacterial growths were detected, respectively (Figure 4). The culture showed groups of gram-positive cocci arranged in chains. Further analysis confirmed the presence of *S. anginosus*. The Kirby-Bauer disk diffusion method for antibiotic susceptibility showed that the organisms were sensitive to linezolid, penicillin, and vancomycin.

The remaining investigations showed hypoalbuminemia, elevated sepsis marker (C-reactive protein (CRP)=57 mg/L), and positive Bence-Jones protein in the urine. The coagulation profile, liver function tests, electrolyte panel, and kidney function tests were all in normal ranges. The serum electrophoresis showed small oligoclonal bands that most probably are associated with infections or autoimmune disorders (Table 2). Ultrasound Doppler on both carotid arteries revealed that no evidence of hemodynamically significant stenosis was identified. Esophagogastroduodenoscopy (EGD) revealed Barrett's esophagus (biopsied), hiatus hernia, bile gastritis, and non-erosive duodenitis. Computed tomography of the thorax, abdomen, and pelvis (CT-TAP) showed no significant findings and no source of bleeding. CT colonography revealed no abnormalities in the colon and no evidence of active colitis.

**Table 2:** Laboratory test result

Parameter	Result	Unit	Reference range
Total protein	65	g/L	60-80
Calculated globulin	42	g/L	-
Total bilirubin	9	µmol/L	<21
Alkaline phosphatase	57	U/L	30-130
Alanine aminotransferase	7	U/L	<41
Magnesium	0.90	mmol/L	0.70-1.00
Phosphate	1.20	mmol/L	0.80-1.50
Sodium	128	mmol/L	133-146
Potassium	4.7	mmol/L	3.5-5.3
Urea	8.0	mmol/L	2.5-7.8
Creatinine (Jaffe)	131	µmol/L	62-106
Iron	2.1	µmol/L	5.8-34.5
Ferritin	488.9	ng/mL	30.0-400.0
Transferrin	1.33	g/L	2.00-3.60
Transferrin saturation	7	%	15-50

The patient was managed with gentamycin 80 mg once and then amoxicillin 2 g/4 hours for five days. Then, he was started on ceftriaxone 2 g once per day for 28 days in addition to the management of IDA that included ferrous sulfate and folic acid. Finally, the patient was discharged from the hospital after confirmation of complete recovery via several culture-negative results.

## Discussion

In our case report, there were two unique findings: firstly, the causative organism of SBE is *S. anginosus* and secondly, the atypical presentation of the patient was symptomatic IDA and progressive weight loss.

*S. anginosus* in SBE

The *S. anginosus* group is part of the normal flora that inhabits the skin and the superficial epithelial layers in the oral cavity, gastrointestinal tract (GIT), and genitourinary tract. They are usually considered harmless commensals that do cause any pathology under normal circumstances. However, upon disruption of the normal mucosal barrier, they gain access to the underlying tissues and the bloodstream and change their physiological habitats, or when the host's immune system is compromised, they turn into opportunistic organisms that are capable of producing different pathologies in the organs of the human body [5]. Studies show that the pathogenic *S. anginosus* group is linked to many pathologies like dental abscesses, intracerebral abscesses, respiratory tract infections, GIT infections, and genitourinary tract infections [4]. Unlike the other species of *S. viridans*, the *S. anginosus* group has the lowest possibility of causing SBE [6]. Woo et al. reported that out of 377 cases diagnosed with infective endocarditis, only six cases have *S. anginosus* group infections [7]. Studies showed that the *S. anginosus* group is less virulent and less associated with abscess formation; this could be explained by the fact that deoxyribonuclease, hyaluronidase, and chondroitin sulfatase enzymes are less active in the *S. anginosus* group in comparison to *S. intermedius* and *S. constellatus* [8]. However, upon studying the effect of the *S. anginosus* group in lab rats, the bacterial densities of SBE and the load of bacteremia were significantly higher among *S. anginosus* in comparison to *S. intermedius* and *S. constellatus*, and this correlation has never been studied among humans so far [9]. In our study, we managed to isolate the *S. anginosus* group from the vegetations of the patient's mitral valve. However, there were no other septic foci through the whole body of our patient. Finn et al. reported a case of SBE caused by *S. anginosus* complicated by multiple liver abscesses in a patient with a history of splenectomy [10]. Chang et al. also managed to isolate *S. anginosus* in a case of community-acquired *S. anginosus* endocarditis on a bicuspid aortic valve in a previously healthy patient [11]. Controversial results have been associated with the true virulence of the *S. anginosus* group and their capability of causing SBE. Some case reports, like the previously mentioned studies, considered *S. anginosus* group endocarditis as an aggressive condition with dangerous intracardiac or extracardiac complications [12,13]. Other studies, including ours, reported *S. anginosus* group endocarditis with less aggressive complications and fewer possibilities of extracardiac or embolic events. Hence, our report highlights the rarity of the mild clinical course observed here. However, out of 5336 cases of infective endocarditis, Escrihuela-Vidal et al. found that *S. anginosus* group endocarditis accounted for 1.4% of the cases and there was no significant difference between *S. anginosus* group and non-*S. anginosus* group endocarditis in the intracardiac or extracardiac complications [14].

IDA as an atypical presentation in SBE

Anemia is a common association with SBE. It usually develops secondary to chronic infection, splenic sequestration of the red blood cells (RBCs), valvular hemolysis, and bone marrow suppression by bacterial toxins. It can also be attributed to hematuria secondary to glomerulonephritis among SBE patients [2,15]. In our study, the patient presented with IDA. His EGD and the CT colonography revealed no signs of active gastrointestinal bleeding or active colitis, respectively. Excluding the possibility of IDA secondary to occult bleeding raises the suspicion of IDA associated with the SBE of the patient. Studies demonstrated that normocytic normochromic hemolytic anemia is associated with SBE caused by *S. bovis*, *Cardiobacterium hominis*, and *Actinomyces israelii*, respectively [16-18]. The mechanism of intravascular hemolysis is well discussed in the literature. It can be mediated by the mechanical shearing of the RBCs by the valve or the superimposed vegetations [19], the sheering stress or splenic sequestration of the RBCs [20], or even the rapid acceleration and collision of the RBCs in patients with patent ductus arteriosus. To the best of our knowledge, this is the first case report with IDA secondary to SBE. Yet, we still have not confirmed the underlying mechanism. Hence, further studies need to be conducted to confirm or reject the relationship and figure out the underlying pathophysiological process.

## Conclusions

The *S. anginosus* group can cause a virulent form of SBE. It can also be complicated by severe mitral regurgitation. This case demonstrated a possibility of the *S. anginosus* group causing infections in a native valve. This infection can be presented by unique manifestations like IDA. Further literature needs to be conducted to study the impact of this group on the valves of the heart and confirm the association between IDA and SBE.
